# The Prevalence of Hepatitis E Virus Infections among Swine, Swine Farmers and the General Population in Guangdong Province, China

**DOI:** 10.1371/journal.pone.0088106

**Published:** 2014-02-10

**Authors:** Huanbin Liang, Shuo Su, Shengchao Deng, Honglang Gu, Fangxiao Ji, Lifang Wang, Chumin Liang, Heng Wang, Guihong Zhang

**Affiliations:** MOA Key Laboratory of Animal Vaccine Development, College of Veterinary Medicine, South China Agricultural University, Guangzhou, China; Virginia Polytechnic Institute and State University, United States of America

## Abstract

Hepatitis E virus (HEV) infection is widespread in China, but few studies have been carried out in Guangdong Province. This study aimed to characterize the prevalence of HEV infections among swine, swine farmers and the general population in Guangdong Province. We conducted an epidemiological study that included swine, swine farmers and health examination attendees in Guangdong from 2011 to 2013. The overall seroprevalence of anti-HEV antibodies in swine was 64.7%. The results revealed that growing pigs, sows and boars (OR ranges from 3.5 to 21.5) have a higher risk than nursery pigs. HEV RNA in swine bile showed that HEV is epidemic in swine in the Pearl River Delta, with the highest prevalence of 22.73% in Foshan. Some genomes of HEV strains from each district were sequenced. Phylogenetic analysis of partial open reading frame 2 (ORF2) shows that they belong to genotype IV and are most closely related to isolates from China. In total, 307 participants were enrolled in the study, including 114 swine farmers and 193 attendees from hospitals. IgG anti-HEV was detected in 48.25% of swine farmers and in 38.34% of the general population. Seroprevalence rates were almost stratified by age, with a higher positive rate for males compared to females across all age groups. Women on swine farms appeared to have a lower risk of infection compared to the general population, revealing that the risk factors for HEV infection are not unique. The results suggested that there were other risk factors for HEV infection. HEV infection is prevalent in Guangdong, but due to the small sample sizes, more investigations are needed to assess the potential impact of HEV infection, and many additional risk factors should be considered.

## Introduction

The hepatitis E virus (HEV) is a small non-enveloped RNA virus that belongs to the genus Hepevirus in the family Hepeviridae [1]. There are 4 HEV genotypes but only 1 serotype. Genotypes 1 and 2 infect only humans and are mainly endemic to developing countries such as those in Asia, Africa and South America. Genotypes 3 and 4 infect humans, pigs and other animal species in America, Europe and Asia. Obviously, the genotypes differ with respect to epidemiological distribution and host species [2], [3].

Hepatitis E virus (HEV) infection is widespread in China [4], [5]. However, few studies have been conducted in Guangdong, and such studies were only for human infections [6], [7]. Those studies also only dealt with prevalence and not risk factors, so consequently, it is necessary to identify risk factors for HEV infection. Consumption of fecally contaminated water has played an important role in hepatitis E epidemics in China [8], [9]. Although evidence gathered suggested that eating raw or inadequately cooked meat and offal from pigs could cause HEV infection [10], [11], this transmission route has not been reported in China. On the other hand, recent studies in China have shown the seroprevalence of HEV in swine, swine farmers and the general population [4], [12]–[15]. Nevertheless, there is a lack of such research in Guangdong Province, China. Meanwhile, an increasing number of HEV infections have been detected in nearby districts, including Hunan [5], Hong Kong [16], and Taiwan [17]. Consequently, additional surveys should be carried out, and we aim to determine the relationship between human and swine HEV infections. Recently, the first hepatitis E vaccine was approved by the State Food and Drug Administration in China [18]. Prevention and control programs that include vaccination would need to specifically target people living in regions with relatively higher prevalence. Thus, it is important to understand the local epidemiology of Guangdong Province.

## Materials and Methods

### Study Area and Recruitment of the Study Population

Swine bile samples and serum samples were collected from 2011 to 2013 from Guangdong Province, China. Two hundred eighty-eight swine bile samples were collected in the Pearl River Delta. Bile samples were collected from nursery pigs (<4 wks), growing pigs (4 wks-6 mos), sows (>7mos) and boars (>7mos). Five hundred sixty-one samples of swine serum and 114 samples of farmers’ serum samples were collected from swine farms located in Guangdong. Thirty-four different farms were sampled, and the information of farms and samples was listed in TABLE 1. Health examination attendees were enrolled at the Third Affiliated Hospital of Sun Yat-sen University. One hundred ninety-three samples of human serum were collected from the attendees at that hospital, which is located in Canton. A questionnaire was also administered to collect relevant information from both the swine farmers and the urban attendees.

**Table 1:** The information of swine farms and samples.

Farm	Bile(positive/total)				Farm	Serum(positive/total)			
	N	G	S	B		N	G	S	B
Dongguan1	1/5	0/7	0/1	1/1	A	4/12	0	26/30	6/7
Dongguan2	0	1/6	1/1	0/1	B	6/13	0	25/33	7/7
Foshan1	0/3	1/5	1/1	1/1	C	1/7	0	18/28	6/6
Foshan2	2/8	1/6	1/2	0	D	4/14	5/12	14/20	2/3
Foshan3	1/7	2/10	0/1	0	E	6/12	3/6	16/18	3/4
Guangzhou1	0/2	1/9	0/1	0	F	2/12	8/18	11/20	4/4
Guangzhou2	0	1/7	0/1	0	G	3/8	9/11	17/24	5/5
Huizhou1	0/2	2/11	0/2	0	H	3/9	7/10	16/22	5/5
Huizhou2	1/5	1/7	0	0	I	5/16	7/8	19/25	6/6
Huizhou3	0	2/13	0	0	J	3/9	5/5	19/19	5/5
Jiangmen1	0/3	1/10	1/1	1/1	K	4/11	4/4	16/22	4/5
Jiangmen2	1/9	2/12	1/2	0	L	5/10	4/6	11/25	4/5
Jiangmen3	1/3	0/5	2/2	0					
Shenzhen1	0/2	0/4	0/1	0					
Shenzhen2	0/2	1/6	1/1	0					
Zhaoqing1	0/6	1/8	0	0					
Zhaoqing2	0/7	1/10	0/1	0					
Zhongshan1	2/6	1/7	0/1	0					
Zhongshan2	1/4	1/5	0/1	0					
Zhongshan3	1/6	2/9	0/1	0					
Zhuhai1	1/6	0/8	1/2	0					
Zhuhai2	1/4	1/4	0/2	0					
Total	13/90	23/169	9/25	3/4		46/133	52/80	208/286	57/62

Bile samples were collected from 22 different swine farms including large-scale farms and family-scale farms in 9 districts of Pearl River Delta. Swine sera were sampled from 12 large-scale swine farm in Guangdong. N, nursery pig; G, growing pig; S, sow; B, boar.

### Ethical Considerations

This study protocol was reviewed and approved by the Institutional Review Board at the Guangdong Center for Disease Control and Prevention. An informed consent form was provided to and signed by each participant. Human sampling procedures were also approved by the Guangdong Center for Disease Control and Prevention.

Pig sampling procedures were approved by the Animal Care and Use Committee of Guangdong Province, China. Our sampling processes were assisted by local authorities and veterinarians. All animal research was conducted under the guidance of the CDC's Institutional Animal Care and Use Committee, and all animal research was conducted in a facility accredited by the Association for Assessment and Accreditation of Laboratory Animal Care International. The animal research in our study was approved by the Guangdong Province Animal Disease Control Center. The contract-numbers of the approval documents from the ethic committees is 2013-02.

### RNA extraction and reverse transcription-nested PCR

Total viral RNA was extracted using Trizol (Invitrogen, Carlsbad, CA, USA) according to the manufacturer’s instructions. We designed two sets of degenerate HEV primers to detect genetically divergent strains (partial ORF2 gene) of HEV, using nest PCR after reverse transcription (RT). The primers HEV-EXF (5'- GAR GCY TCT AAT TAT GCY CAG TA -3') and HEV-EXR (5'- AAA GCC ARA GCA CAT CAT TAG C -3') were used as external primers, and HEV-INF (5'- TTG GCG CTC RGT TGA GAC CTC -3') and HEV-INR (5'- TRG CTA TAC CCT TRT CCT GCT G -3') were used as internal primers. The PCR profile for both rounds was as follows: 94°C for 5 min, 30 cycles at 94°C for 30 s, 56°C for 30 s, and 72°C for 45 s, and then a final extension at 72°C for 10 min. A negative control (water) and a positive control (HEV RNA) were included in each RT and PCR run. Furthermore, Primer Premier 5.0 was used to design primers to amplify the genes of HEV.

### Detection of antibodies to HEV in serum

To detect immunoglobulin G (IgG) against HEV (anti-HEV), a commercial enzyme-linked immunosorbent assay (ELISA) from Wantai Biopharmaceutical, Inc. (Beijing, China) was used, and laboratory analysis was carried out according to the manufacturer’s instructions [17], [19]-[20]. The kits for detection of antibodies to HEV in serum differ for swine and human. For swine, a sandwich antigen based ELISA was used to detect the total antibodies; but for human, an indirect method was used to detect only the IgG-class antibodies.

### Sequencing and phylogenetic analysis

**Figure 1:**
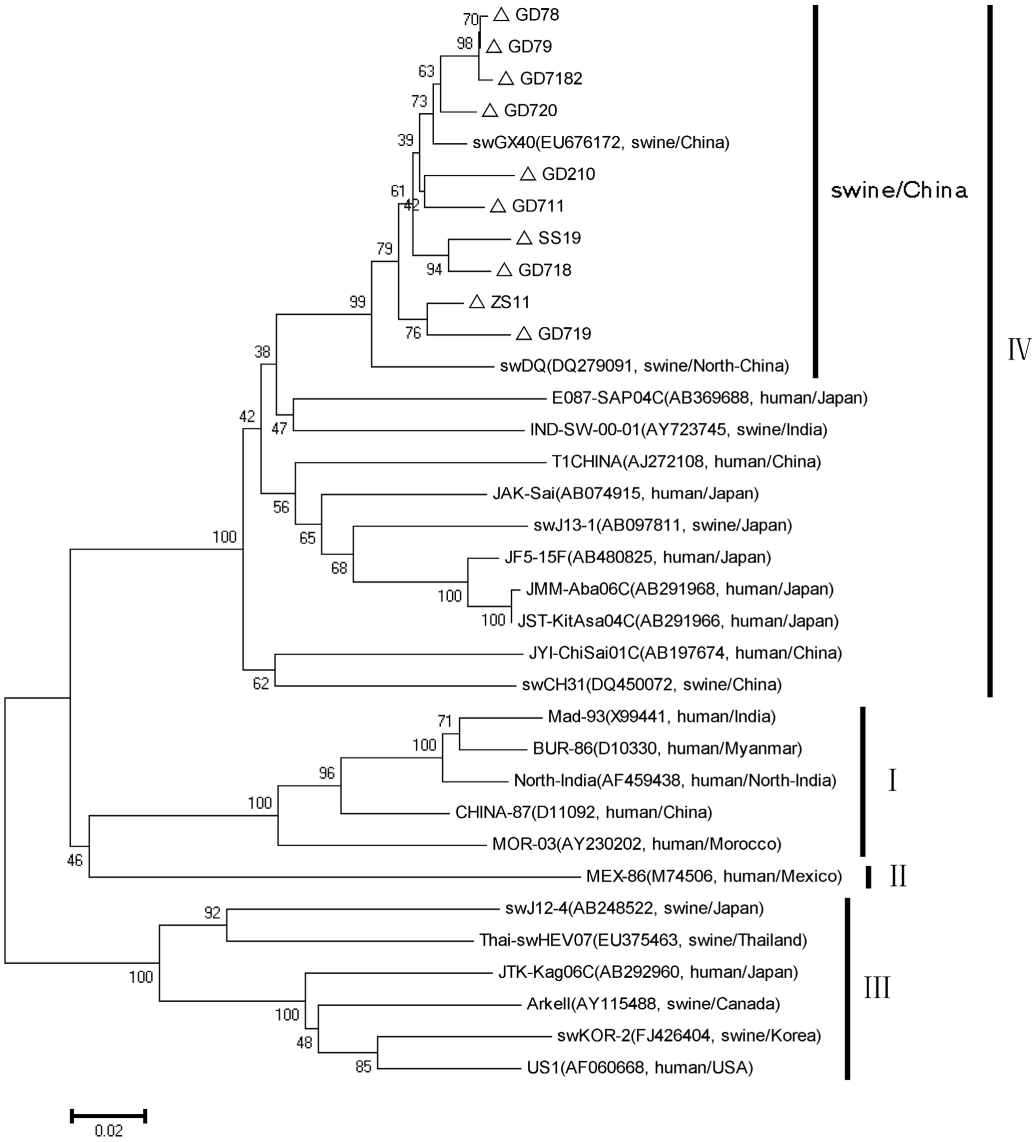
A phylogenetic tree based on the full nucleotide sequence of HEV. Total RNA positive bile samples are 48, from 9 different districts in Guangdong. We have chosen at least one sample from each district by random sampling. The amplification products of ORF2 (509 nucleotides, primer sequences were HEV-INF and HEV-INR as described above) from 10 positive bile samples were sequenced and compared. The nucleotide sequence identity among the 10 swine HEV isolates obtained from pigs from different farms in three years ranged between 94.3 and 99.8%. Phylogenetic trees were constructed by the neighbor-joining method based on the partial nucleotide sequence of the ORF2 region (509 nucleotides). The bootstrap values (expressed as percentages) were determined on 1000 re-samplings of the data sets. △were the isolates in this study.

The PCR products of the second round were purified (Invitrogen) and then ligated into pMD18-T vectors (Takara Dalian Co. Ltd., China). The plasmids were extracted, and the inserts were sequenced at Life technologies (Shanghai, China). Ten sequences isolated in this study were deposited in GenBank under accession number KJ001824-KJ001832 and JX855794. Percent identity was calculated with Lasergene (DNAstar Version 7.1.0). Genetic distances between the known strains and the virus isolates were calculated with the MEGA software (Version 5), and phylogenetic trees were constructed by the neighbor-joining method. Twenty-five related HEV strains were used as references in Fig 1.

### Statistical Analysis

The chi-square test was used to analyze categorical variables when appropriate. The magnitude of the association between variables and seropositivity is expressed as an odds ratio (OR) with 95% confidence intervals (95% CI). Univariate and multivariate logistic regression analysis was carried out to identify which variables were significantly associated with anti-HEV seropositivity. Logistic regression was performed using R (R version 2.15.3 for windows x64). The results of the OR and its 95% CI were prepared using R with a Fourfold plot [21]. A P value of less than 0.05 was considered significant. The best fit trendline for the positive rate data was selected based on the R-squared value of the curves drawn using the Microsoft Office Excel 2007 program (Microsoft, Redmond, WA). Questionnaire data collected from farmers and urban attendees were digitized and verified.

## Results

### The prevalence of antibodies against HEV among pigs

**Figure 2:**
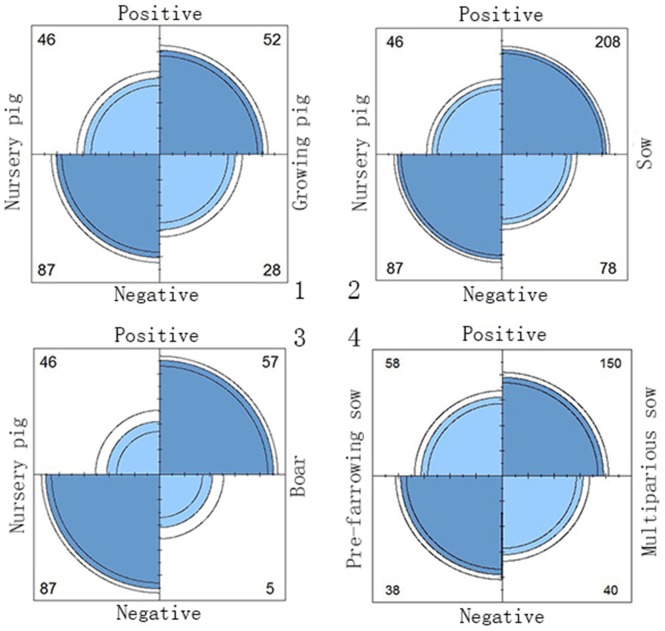
Fourfold plot for odds ratio (OR). Univariate analysis results are presented in the Fourfold plot diagram. Fourfold display for seropositive data of sows: Evidence for pig herds bias or multiparity bias? The area of eachshaded quadrant shows the frequency, standardized to equate the margins for different herds and seropositive. Circular arcs show the limits of a 95% confidence interval for the odds ratio. 1. Compared with nursery pigs, the OR of growing pigs was 3.51(95%CI, 1.95–6.33; p<0.0001); 2. Compared with nursery pigs, the OR of sows was 5.04(95%CI, 3.27–7.77; p<0.0001); 3. Compared with nursery pigs, the OR of boars was 21.56(95%CI, 9.47–49.10; p<0.0001); 4. Compared with pre-farrowing sows, the OR of multiparous sows was 2.46(95%CI, 1.45–4.18; p<0.01).

Among the 561 swine serum samples collected from swine farms, the overall seroprevalence was 64.7% (363/561). Swine sera were divided into 4 groups according to the pig herds on the farms. The positive rate of HEV-specific IgG in all groups ranged from 34.59% to 91.93% (TABLE 2). The data show some possible risk factors for HEV infection and revealed that boars had an approximately 21.5-fold higher risk (odds ratio [OR], 21.56; 95% confidence interval [CI], 9.47–49.10; p<0.0001), sows had a nearly 5-fold higher risk (OR, 5.04; 95%CI, 3.27–7.77; p<0.0001) and growing pigs had a 3.5-fold higher risk (OR, 3.51; 95%CI, 1.95–6.33; p<0.0001) than nursery pigs. Sows were divided into two groups to determine if the risk factor for HEV infection was due to multiparity. The results showed that multiparous sows had an approximately 2.5-fold higher risk (OR, 2.46; 95%CI, 1.45–4.18; p<0.01) than pre-farrowing sows, and there were statistically significant differences between them (Fig 2).

**Table 2:** The prevalence of antibodies against HEV among pigs in 2011–2013, Guangdong Province, China.

	Serum sample		Univariate analysis		
	Positive/Total (n)	Positive rate (%)	OR^a^ (95% CI^ b^)	X^2 c^	P value
Different swine farm					
Nursery pig (<4 wks)	46/133	34.59	ref^d^		
Growing pig (>4 wk-6 mos)	52/80	65.00	3.51(1.95–6.33)	17.40	<10^4^
Sow (>7 mos)	208/286	72.72	5.04(3.27–7.77)	53.74	<10^4^
Boar (>7 mos)	57/62	91.93	21.56(9.47–49.10)	53.53	<10^4^
Sow					
Pre-farrowing sow	58/96	60.42	ref		
Multiparous sow	150/190	78.95	2.46(1.45–4.18)	10.13	<0.01

a: OR, odds ratio;

b: 95%CI, 95%confidence interval;

c: X^2^, Chi-Square Test;

d: ref, reference.

### HEV RNA detection in pig bile samples and phylogenetic analysis

**Figure 3:**
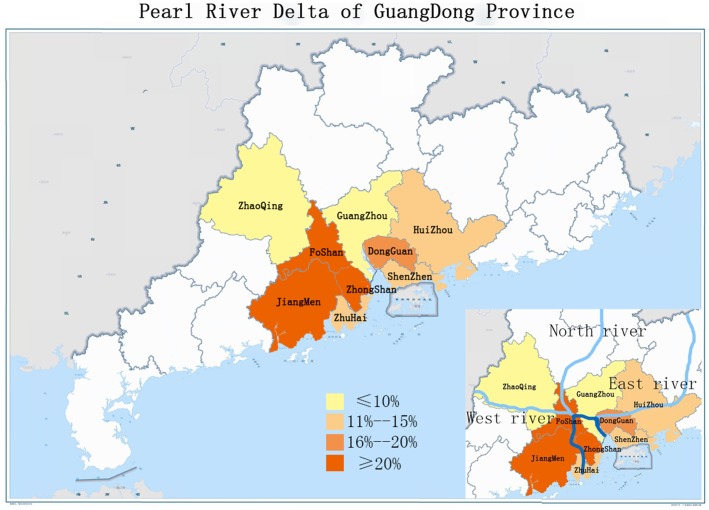
A map indicating the prevalence of HEV RNA in the Pearl River Delta of Guangdong Province.

The 228 pig bile samples that were collected from 9 districts of the Pearl River Delta had been stored in Guangdong since 2011. As shown in TABLE 3, the detection rates of HEV RNA varied among districts of Guangdong Province. The highest prevalence was 22.73% (95%CI, 2.5–33.86) in Foshan, and the lowest was 6.25% (95%CI, 0–14.64) in Zhaoqing. The prevalence of HEV RNA in Guangzhou and Shenzhen ranged from 10% to 12.5%, while those in Huizhou, Zhuhai, and Dongguan ranged from 15% to 18.18%. Like Foshan, Jiangmen and Zhongshan also had a high prevalence of over 20% (Fig 3).

**Table 3:** The prevalence of HEV RNA in pig bile samples collected from the Pearl River Delta. b

District	No. of bile samples	No. of positive HEV RNA samples	Analysis	
			positive rate (%)	95% CI
Dongguan	22	4	18.18	2.5–33.86
Foshan	44	10	22.73	10.35–35.11
Guangzhou	20	2	10.00	0–23.14
Huizhou	40	6	15.00	3.93–26.07
Jiangmen	48	10	20.83	9.34–32.32
Shenzhen	16	2	12.50	0–28.70
Zhaoqing	32	2	6.25	0–14.64
Zhongshan	40	8	20.00	8.24–31.76
Zhuhai	26	4	15.38	1.51–29.25

b Pig bile samples have been collected from 9 districts of the Pearl River Delta since 2011.

Phylogenetic tree constructed by the neighbor-joining method based on the partial nucleotide sequence of the ORF2 region (509 nucleotides) of 34 HEV isolates. Ten isolates were compared with 24 HEV reference sequences of different genotypes to analyze the genetic characteristics and evolutionary regularity. The nucleotide sequence identity among the 10 swine HEV isolates obtained from pigs from different farms in three years ranged between 94.3 and 99.8%. Based on the phylogenetic and homology analysis, the isolates we obtained from the 9 different districts shared high nucleotide similarities, and they were all belonged to genotype IV HEV. The isolates also shared the same phylogenetic branch with other Chinese isolates. The sequence similarity was approximately 87% between the isolates and the other genotype IV strains that had been isolated from HEV patients in China and Japan (Fig 1).

### HEV-specific IgG detection in humans

The seroprevalence of IgG anti-HEV in serum samples collected from swine farmers was almost as high than that of the general population. However, it was surprising to find that the positive rates in urban people (38.34%, 74/193) were very high and almost as high as the total prevalence found in swine farm workers (48.25%, 55/114).

Univariate analysis was used to compare HEV seroprevalence rates between swine farmers and urban attendees. As seen in TABLE 4, the overall positive rates for gender (p>0.23) and exposure to pigs (p>0.05) appeared to have weak associations with HEV seropositivity. However, when age was stratified into groups, differences were visualized using the best fit trendline. Samples of ages ≤29, 30–39, 40–49, 50–59, and ≥60 years were examined (TABLE 4). There were significant differences between the age groups, and anti-HEV was distributed among all age groups. Seroprevalence rates stratified by age are shown in TABLE 4. Without any age group showing a plateau, the best fit curves showed that the positive rate for HEV IgG increased steadily with age (Fig 4.1–4.3). The trendlines suggested a higher positive rate for males than females across all age groups, especially among the swine farmers (Fig 4.1–4.3). The trendline of swine farmers in the 40–49 age group had a steeper slope (OR, 4.14; 95%CI, 2.15–7.96) (Fig 4.4–4.6). Nevertheless, it should be noted that women from swine farms appeared to have a lower risk of infection compared to that of the general population (Fig 4.5).

**Figure 4:**
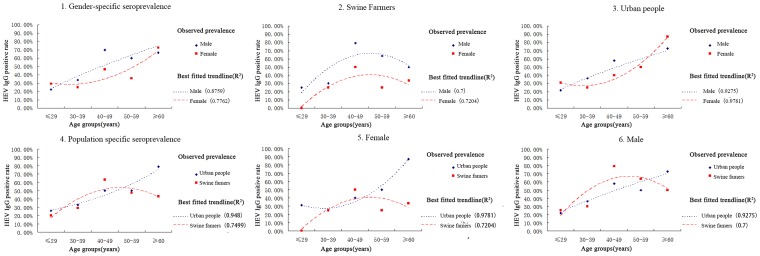
The estimated seroprevalence of the hepatitis E virus in Guangdong Province. The best fit trendline for the positive rate data was selected based on the R-squared value of the curves drawn using the Microsoft Office Excel 2007 program (Microsoft, Redmond, WA). 1. Overall gender-specific seroprevalence; 2. Gender-specific seroprevalence of swine farm; 3. Gender-specific seroprevalence of the general population; 4. Overall population-specific seroprevalence; 5. Population-specific seroprevalence of females; 6. Population-specific seroprevalence of males.

**Table 4:** The anti-HEV rates of the population in Guangdong province, China, according to age, gender, and pig-exposure status.

	Sample		Univariate analysis			
	Positive/Total (n)	Positive rate (%)	OR^a^ (95% CI^b^)	X^2 c^	P value	
Age						
≤29	22/88	25.00	ref^d^			
30–39	24/76	31.58	1.38(0.60–3.21)	0.58	0.45	
40–49	51/88	57.95	4.14(2.15–7.96)	18.35	<10^4^	**
50–59	14/29	48.28	2.80(1.09–7.22)	4.51	<0.05	*
≥60	18/26	69.23	6.75(2.60–17.54)	15.35	<10^4^	**
Gender						
UF	30/77	38.96	ref			
UM	44/116	37.93	0.96(0.60–1.54)	0.03	≈1	
FF	19/47	40.43	ref			
FM	36/67	53.73	1.71(0.72–4.08)	1.46	0.23	
Pig-exposure						
UF	30/77	38.96	ref			
FF	19/47	40.43	1.06(0.54–2.08)	0.03	≈1	
UM	44/116	37.93	ref			
FM	36/67	53.73	1.90(0.99–3.66)	3.69	0.05	

a: OR, odds ratio; b: 95%CI, 95%confidence interval; c: X^2^, Chi-Square Test; d: ref, reference; UF, urban female; UM, urban male; FF, swine farm female; FM, swine farm male; **, significant difference; *, different.

## Discussion

### Seroprevalence and risk factor of swine HEV in Guangdong

Some previous studies have been given a preliminary overview of the seroprevalence situation of swine HEV in China. A previous serological investigation found that the seroprevalence of anti-HEV IgG in pig herds was 26.8% (22/82) [22]. Another survey revealed that the prevalence of swine HEV was as high as 91.7% (77/84) [23]. The present study showed that a great HEV infection pressure exists in Guangdong pig herds, which had an overall seroprevalence of 64.7% (363/561), with an even higher positive rate in sows (72.72%, 208/286) and boars (91.93%, 57/62). The higher infection pressure on farms may have increased the probability of infection and consequently reduced the period between exposure and infection as described by Bouwknegt et al. [24]. With long-term exposure, the seroprevalence of swine HEV obviously accumulated with age. As the most senior animals on the farms, boars had the highest risk compared with piglets (approximately 21.5-fold) (Fig 2). In the present study, the HEV seroprevalence among multiparous sows and pre-farrowing sows was found to be 60.47% and 78.95%, respectively. According to a previous study conducted by Casas et al. [25], pre-farrowing sows had a relatively low seropositive (17%). These discrepancies might be caused by the different sensitivities of ELISA kits and variations. This study indicated that multiparous sows had an approximately 2.5-fold higher risk (OR, 2.46) than pre-farrowing sows. The reasons are still not clear. Recent studies revealed that stress from farrowing or high levels of steroid hormones from the physiological changes during pregnancy might reactivate HEV replication [26], [27]. However, risk factors for HEV infection could be diverse. Not only pregnancy but also age could be a possible risk factor. Much more study should be progressed to figure out.

### Comparison of human HEV prevalence between Guangdong and other provinces in China

In China, many surveys have shown an irregular seroprevalence of HEV in several provinces. Only approximately 10–20% of people have had an HEV infection in the Jilin province of China [15], while prevalence in northeastern China (47.7%–50%) has been reported to be very high [28]. However, such differences could have arisen because of differences in the study sample sizes or might reflect true regional differences. In the current survey, compared with the seroprevalence of HEV in the adjacent districts of Hunan (22.4%, 488/2181) and Hong Kong (28.7%, 129/450), Guangdong (38.3%, 74/193) had a significantly higher positive rate [5], [16]. Guangdong rates were comparable to those in Taiwan (12%) and Korea (23.1%) but less than that in the highly endemic southwest France (52.5%), where the same diagnostic assay was used [17], [29]–[30]. Nevertheless, direct comparisons of studies were limited by the use of different diagnostic assays [20]. The assay kit (Wantai) used in the current study, which had been used for a survey in Taiwan, is more sensitive than the HEV diagnostic serology kits previously available [17]. As Guangdong is a developed and populous area in China, its high population density, frequent public events, and abundant water resources in some regions of this province may contribute to its higher endemicity.

### Risk factors for human HEV infection among genders and ages

The present study indicates the same conclusion as has been reported in several previous surveys [5], [16]–[17], which is that the positive rate for HEV IgG increased with age (TABLE 4) and that males commonly had higher rates than females, as shown in Fig 4.1–4.3. The significantly higher seroprevalence of HEV on swine farms compared to the general population had also been reported in several countries [31]. However, the trend is the opposite in Denmark [32] and northern Thailand [33]. According to Fig 4.4–4.6, the trendline for swine farmers showed a steeper slope in the 40–49 age group, revealing that occupational exposure could be a strong risk factor for HEV infection. Occupational exposure to pigs may be a relevant risk factor, but it is not the only risk factor for HEV infection. The higher prevalence of HEV infection in males than in females is even more obvious on swine farms. In the present study, the positive rates of urban people (38.34%, 74/193) were very high and very close to the total prevalence of swine farm workers (48.25%, 55/114). Although in contrast to urban people, swine farm workers came in contact with infected pigs in closer intervals. In addition, an increase in the HEV seroprevalence was noted in swine farmers of advanced age, while that of the general population remained on an upward trend across all ages (Fig 4.4–4.6). This is not surprising because several risk factors contributed to the seroprevalence of HEV in the general population. Validation of this trend using a larger cohort is required.

These results may be associated with the complicated exposure factors in cities and the single exposure factor on swine farms. The positive rate for urban females is higher than the positive rate for farm females, which confirms this conjecture (Fig 4.5). This may induce a similar seroprevalence between different genders and may be responsible in part for the high seroprevalence of the general population in both genders. Due to low cultural awareness and a lack of personal hygiene knowledge, workers on the swine farms in Guangdong are ill-equipped to protect themselves from HEV infection. The seroprevalence of females on swine farms is obviously lower than that of males and even lower than the overall seroprevalence of the general population (Fig 4.2 and Fig 4.5). Altogether, these findings may explain why the seroprevalence of urban females is slightly higher than those working on farms.

### Elementary molecular epidemiology survey of swine HEV in Guangdong

In the current study, a total of 16.67% of pigs had HEV RNA in their bile. Nonetheless, this prevalence was significantly higher than that found in the Netherlands [34], the United States [35], Spain [25], and especially Japan [36]. The contact between workers and infected pig livers could be a possible route of HEV transmission [37], [38]. Water sources contaminated with swine feces may have facilitated HEV transmission between farms [8], [25]. As shown in Fig 3, the Pearl River consists of 3 tributaries, which are named the North River, the East River, and the West River (Fig. 3). The three branches intersect in Foshan (22.73%, 10/44) from 3 different directions and then end up in the Southern Sea of China through the Jiangmen (20.83%, 10/48) and the Zhongshan (20%, 8/40) districts. This reveals that the prevalence of swine HEV in each district accumulated along the Pearl River. These results strongly imply that swine HEV epidemic in Guangdong might mainly be water borne. However, some water samples collected from different districts in Guangdong have been tested for HEV RNA, nothing was found by this time (data not shown). Many more studies should be carried out to determine if a virus could transmit through contaminated river water.

The identity and relationship of HEV isolates in Guangdong with other isolates were shown in this study. In the U.S., HEV isolates from the same geographic region showed higher percentages of similar identities to each other than with those from different geographic regions [39]. In the present study, we found that ten new isolates belong to genotype IV and share the same phylogenetic branch as other Chinese isolates (Fig 1). They all displayed high homology with the swine HEV strains swDQ and swGX40 and exhibited a high identity with the human strains E087-SAP04C that were originally isolated from Japan. In other words, the results revealed that the genotype IV HEV strain currently exists in the pig herds of Guangdong Province. Pork safety should be of great concern for the public health in Guangdong Province.

In summary, this survey shows the possible relationship among swine, swine farmers and the general population in Guangdong Province, China. Exhaustive reports and epidemiological investigations in Guangdong were carried out, and the results highlighted pork safety concerns. Other risk factors for HEV infection were implied in this study. Meanwhile, the prevalence of HEV in Guangdong could provide a reference for vaccinations. However, there were some limitations to this study. The sample size was relatively small and may not be fully representative of Guangdong. Many more surveys should be carried out to confirm these findings. Thus, an extensive public health surveillance system needs to be established and maintained in Guangdong Province.

## Supporting Information

Fig. S1 Analysis of nucleotide identity of partial ORF2 among 10 HEV isolates and with represent HEV isolate.
(TIF)
