# Supplementary figures and images for: The Prevalence of Hepatitis E Virus Infections among Swine, Swine Farmers and the General Population in Guangdong Province, China
Source: PLoS One. 2014 Feb 10;9(2):e88106. doi: 10.1371/journal.pone.0088106 (PMC3919735; doi:10.1371/journal.pone.0088106)

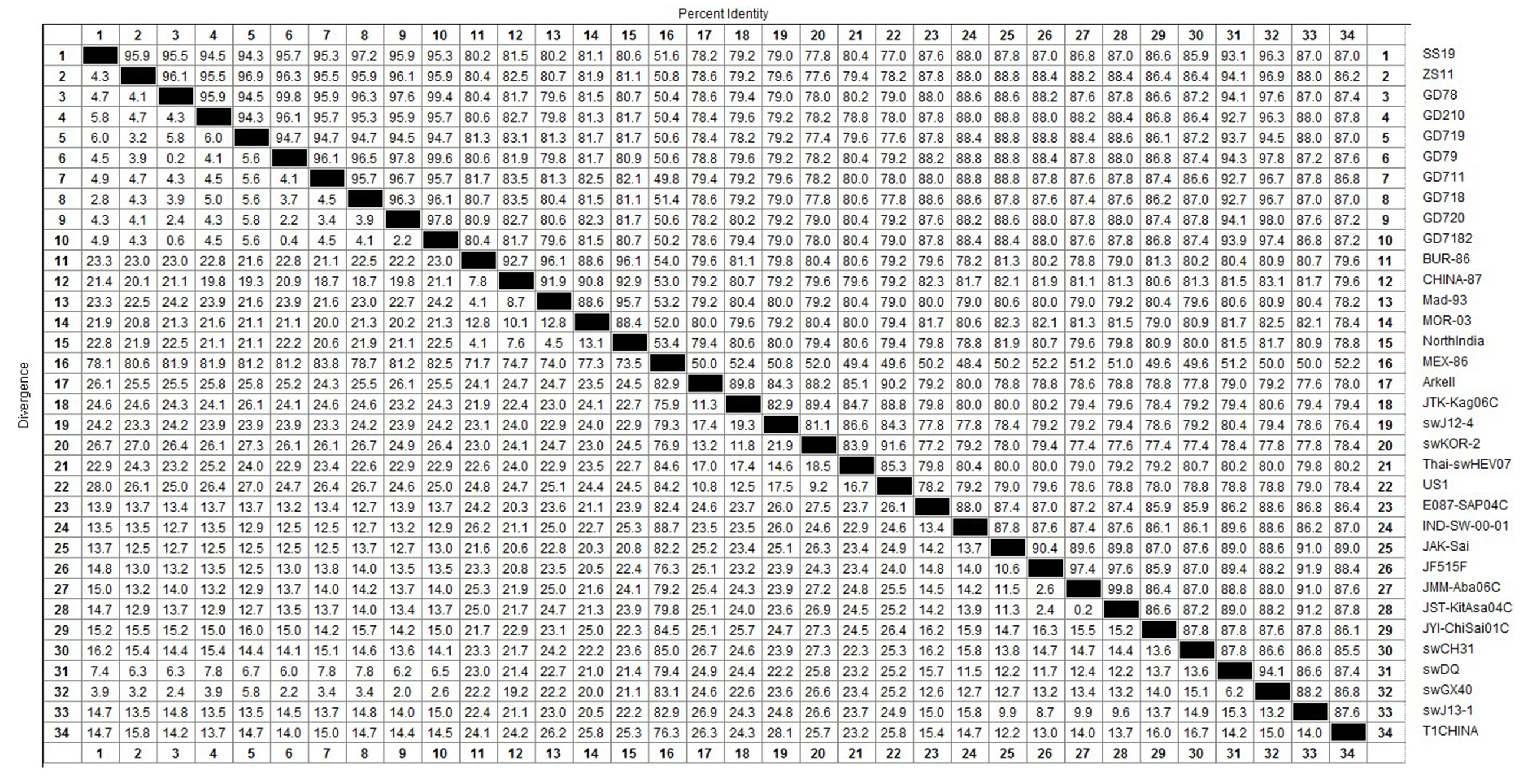

Supplement: Fig. S1 — Analysis of nucleotide identity of partial ORF2 among 10 HEV isolates and with represent HEV isolate. (TIF) [file pone.0088106.s001.tif]
